# Application of the Weathering Framework: Intersection of Racism, Stigma, and COVID-19 as a Stressful Life Event among African Americans

**DOI:** 10.3390/healthcare9020145

**Published:** 2021-02-02

**Authors:** Fathima Wakeel, Anuli Njoku

**Affiliations:** 1 College of Health, Lehigh University, 1 W. Packer Ave., STEPS Building, Room 366, Bethlehem, PA 18015, USA; 2 Department of Public Health, College of Health and Human Services, Southern Connecticut State University, 144 Farnham Avenue, New Haven, CT 06515, USA; njokua3@southernct.edu

**Keywords:** health disparities, COVID-19/coronavirus, stigma, race/ethnicity, social determinants of health, Blacks/African Americans, racism, systemic racism

## Abstract

The disproportionate impact of coronavirus disease 2019 (COVID-19) on African American communities necessitates an increased focus on the intersectional roles of racism, stigma, and other social determinants of health in influencing disease and mortality risk. The Weathering Framework is applied to demonstrate the dynamic interrelationships between these factors and to conceptualize COVID-19 as a stressful life event that will have profound health implications over the life course for African Americans. Recommendations for population health research, interventions and policies aimed at reducing COVID-19 incidence and mortality, and mitigation of the long-term impacts of the pandemic on communities of color are discussed.

## 1. Introduction

The “coronavirus disease 2019” (COVID-19), a pandemic of lower respiratory tract disease caused by severe acute respiratory syndrome coronavirus 2 (SARS-COV-2) infection and resulting in severe illness and potential death from pneumonia-like symptoms, has significantly beset communities across the world [1,2,3]. As of 16 December 2020, there are over 73.9 million cases of COVID-19 and over 1.6 million COVID-19-related deaths globally [4,5]. The United States (U.S.) has the most COVID-19 deaths, at over 311,000 people, along with over 17.1 million cases and over 10 million recovered patients [5,6]. Persons at higher risk for COVID-19 include adults of any age with certain underlying medical conditions such as cancer, chronic kidney disease, heart conditions, weakened immune system, obesity, smoking, type 2 diabetes mellitus, pregnancy, and sickle cell disease [7]. In addition, African Americans, compared with all other racial/ethnic groups, are more likely to contract COVID-19 and die of the disease [8,9,10]. Data from the Centers for Disease Control and Prevention (CDC) [11] show that compared to non-Hispanic Whites, deaths are 2.8 times higher among Black/African Americans, 2.6 times higher among American Indian or Alaska Native, non-Hispanic persons, 2.8 times higher among Hispanic/Latino persons, and 1.1 times higher among Asians.

While predisposition to underlying health conditions, such as hypertension, diabetes, high blood pressure, and asthma, play a role in COVID-19 racial disparities, a history of exposure to marginalization, discrimination, and racial trauma resulting from a “hierarchy of citizenship” [12], p.2 and subsequent unequal “citizenship in practice” [12], p.5; systemic barriers such as systematic racism within the healthcare system; likelihood of being uninsured; reduced access to affordable medical testing; and denied access to COVID-19 testing, diagnosis, and management also likely contribute to poor COVID-19 health outcomes among African American populations [12,13,14]. Therefore, the disproportionate impact of COVID-19 on African American communities necessitates an increased focus on the intersectional roles of racism, stigma, and other determinants of health in influencing disease and mortality risk. In this paper, we contribute to the literature by proposing an integrated Weathering Framework to demonstrate the dynamic interrelationships between these factors, and to conceptualize COVID-19 as a stressful life event that will have profound health implications over the life course for African Americans.

## 2. Racism and Health 

Over the past few decades, researchers and practitioners have identified racism as a pervasive population health crisis and a root cause of disease in the U.S. and globally. Studies have extensively demonstrated the multifaceted relationships between racism—specifically cultural, interpersonal, and structural racism—and health outcomes among African Americans. At the broadest level, cultural racism, which entails the denigration of a minority group through derogatory and exclusionary stereotypes, imagery, media, and social norms, has been shown to impact health through four key pathways, including stereotype threat, internalized racism, interpersonal racism, and structural racism [15,16,17]. Stereotype threat, defined as “anxieties and expectations that can be activated in stigmatized groups when negative stereotypes about their group are made salient” [17], p. 111, has been associated with an impaired patient–physician relationship, engagement in unhealthy behaviors, reduced compliance with treatment recommendations, higher blood pressure, and weight gain among minority groups [18]. Further, internalized racism, which is when individuals in minority groups implicitly accept negative stereotypes about the group, has been linked to decreased psychological wellbeing and increased risk of alcohol misuse, depression, and obesity [19]. It must be noted that both stereotype threat and internalized racism are likely inherited across generations among minority groups through the transmission of social norms and familial expectations. 

Interpersonal racism is another mechanism through which cultural racism is related to adverse health outcomes. Interpersonal racism, though sometimes demonstrated through egregious behaviors such as hate crimes, police brutality, or macroaggressions, is more often rooted in implicit (or unconscious) bias and can manifest during routine social interactions as microaggressions, increased vigilance in public settings, reduced likelihood of obtaining patient-centered care from healthcare providers, and inequitable assessments by instructors or employers. [20,21]. It is important to note that one can be exposed to interpersonal racism either directly or indirectly through the lived experiences of others [22]. In addition to serving as an acute traumatic stressor, especially in the case of egregious or violent behaviors, interpersonal racism also operates as a chronic stressor [23,24]. Widespread research has indicated that exposure to chronic stressors, such as interpersonal racism, can significantly increase one’s risk of premature mortality and morbidity directly through neuroendocrine (i.e., frequent elevation of one’s neuroendocrine responses and resulting increases in cholesterol, blood sugar, triglycerides, and blood pressure) or immune (i.e., impairment of the immune system and subsequent risk of infection) pathways, as well as indirectly through unhealthy behaviors such as poor diet, smoking, alcohol and other substance misuse, risky sexual behaviors, reduced use of preventative services, and decreased compliance with healthcare recommendations [25,26,27,28,29,30,31,32,33,34,35]. Further, chronic stress has been linked to the development of mental health conditions, such as depression and anxiety, which are also inextricably interrelated with physical health outcomes [33,36,37]. Importantly, stress resulting from cumulative exposure to interpersonal racism can also be transmitted intergenerationally, in that stress, in terms of both perceived stress and stressful life events, before and during pregnancy have been strongly linked to adverse obstetric outcomes, such as pregnancy complications, low birthweight, and preterm birth, which, in turn, have profound and multifactorial implications for long-term maternal and child health outcomes [38,39,40,41,42,43,44,45,46]. 

Structural racism is considered the most insidious form of racism due to its systemic, multifaceted, and multi-generational impacts on health outcomes among communities of color. Structural racism has been defined as “policies, laws, and regulations that systematically result in differential access to services and opportunities in society based on race” [20], p. 2. At the core of structural racism may be the “colour-graded hierarchy of citizenship” [12], p. 2, whereby Whiteness fundamentally defines citizenship, and minority groups, such as African Americans, are not socially regarded as equal to Whites, despite nativity status. A potential product of this phenomenon is that U.S.-born Black mothers experience significantly worse obstetric outcomes, such as preterm birth, low birthweight, and infant mortality, when compared to foreign-born Black mothers living in the U.S. [47,48,49]. This evidence suggests that due to this implicit hierarchy of citizenship, the social, economic, and psychological ramifications of growing up Black in the U.S., as opposed to genetic makeup, places African American communities at higher risk for adverse health outcomes.

Structural racism impacts health through its historical and contemporary influences on equitable access to, and quality of, key social and environmental determinants of health. For example, the practice of redlining by the Home Owner’s Loan Corporation (HOLC) in the 1930s hindered communities of color from obtaining home mortgages and subsequently, access to public transportation, healthcare, and supermarkets, and has played a vital role in engendering residential segregation in the United States [50,51,52,53]. In turn, in communities characterized by widespread residential segregation, African Americans are more likely to live in neighborhoods that have higher levels of poverty, experience reduced access to employment, nutritional, transportation, healthcare, and educational resources and opportunities, and reside in health-inhibiting physical environments [52,54,55,56,57,58]. Beyond the damaging impacts of residential segregation, structural racism has been linked with reduced access to, and quality of, healthcare as well as discriminatory incarceration practices that disadvantage African Americans [50]. 

Further, as discussed with the example of redlining and its long-lasting implications, the social and health inequities resulting from structural racism are transferred across generations. A related example of how structural racism is transmitted intergenerationally is the inheritance of poverty among African Americans, due to greater exposure to factors such as fewer parental material resources, higher likelihood of an unstable family structure, residential segregation, and fewer neighborhood and community-level resources, all of which originate from the insidious legacy of slavery in the U.S. [59,60,61]. Further, the intergenerational transmission of risk attributable to structural racism is perhaps most concretely manifested through the increased risk of adverse obstetric outcomes and higher infant mortality rates in African American communities. For instance, research has shown that African American women exposed to residential segregation are more likely to experience adverse birth outcomes, even after controlling for individual and neighborhood-level poverty [58,62,63,64]. 

## 3. Racism and COVID-19

The COVID-19 pandemic highlights the discrimination and racism that have long contributed to adverse emotional, mental, and physical health outcomes in African American communities [65]. For example, COVID-19 illuminates medical mistreatment and mistrust in the African American community. Members of racial and ethnic minority groups tend to receive lower quality of care than Whites, contributing to poorer COVID-19 outcomes among African Americans [66]. There have been high-profile cases of denied access to COVID-19 testing among African Americans. A beloved 30-year-old African American teacher died of COVID-19 after twice being denied a COVID-19 test [67]. In another tragedy, a nurse infected on her job of 31 years died after being turned away four times with COVID-19 symptoms [68]. Another man sick with COVID-19 was turned away by three hospitals before he died at home [69]. The death of a high-risk man sent home from a hospital and told to call his doctor to schedule a COVID-19 test highlights testing barriers for Black residents [70]. Research has shown that despite being at increased risk of exposure to the virus and requiring more intensive care at the time they tested positive for COVID-19, people of color, particularly African Americans, do not have markedly higher testing rates and face increased barriers to care [71,72].

Moreover, access to COVID-19 testing may depend on where one lives. One investigation found that in four cities in Texas, testing centers were disproportionately located in White communities compared to communities with predominantly Black persons [73]. In other instances, officials have been slow to make testing facilities available and accessible to people living in exclusively Black neighborhoods [74,75]. These factors contribute to greater demand, longer wait times for testing, and increased travel time to testing sites [76,77]. Therefore, African Americans are particularly susceptible to COVID-19 exposure due to disparities in access to care. 

Furthermore, the known risk factors for COVID-19 complications need to be examined within the context of adverse social determinants of health that put minority communities at increased risk for disease and mortality. Such factors include, but are not limited to, reduced access to healthy food, housing density, income, education level, occupation, and crowding conditions [9]. The disparate racial impact of COVID-19 also manifests through African American workers facing more economic and health insecurity from COVID-19 than White workers. Patterns of racism and discrimination mean that African Americans have been more likely to be exposed to the virus through work, and less likely to have access to high-quality health care and the resources, such as health insurance, to maintain their health. Racial discrimination in the labor market means that African Americans are more likely to be paid less, overrepresented in jobs that cannot be done from home, terminated, unemployed longer, and to have their unemployment claims denied, compared to their White peers [78]. Effects of the pandemic on African American workers include devastating job losses, spiking unemployment rates, and increased likelihood to be in front-line jobs as essential workers [79].

## 4. Social Stigma and COVID-19

The unparalleled COVID-19 pandemic has intensified the stigma, marginalization, and structural racism that was already devastating African American communities [80]. Stigma has been described as an experience of individuals or communities who are purposely or inevitably excluded from full social approval or who have something unusual or unpleasant exposed about their moral status [81]. In the context of health, stigma can be described as “the negative association between a person or group of people who share certain characteristics and a specific disease” [82]. Stigma is associated with a lack of knowledge about how COVID-19 spreads, fears about disease and death, a need to blame someone, and gossip that spreads rumors and myths. Stigma can also lead to stereotyping, discrimination, labeling, and other negative behaviors toward others [83].

Discriminatory behaviors such as refusal to deliver service, isolation, bullying, and harassment may be experienced by the stigmatized group along with family members, caregivers, those in the same community, or those of the same racial/ethnic group. Such behaviors may obstruct efforts to mitigate disease. This can result in not getting tested due to negative encounters with the healthcare system and not practicing healthy behaviors, such as social distancing, due to lack of paid time off work [84]. Negative healthcare experiences can breed medical mistrust and lead to hesitancy among African Americans to get the COVID-19 vaccine [85]. Discrimination can thwart healthy behaviors if African Americans fear raising police’s suspicions for wearing a mask or having the police called on them for not wearing one [86,87]. Perception of COVID-associated discrimination has been associated with race/ethnicity and wearing masks [88]. Moreover, preliminary data show that African Americans are disproportionately arrested for social distancing violations [89], increasing the probability of exposure to the stressor of police presence and potentially deadly encounters [86]. These lived experiences of racism, stigma, and COVID-19 further indicate the hierarchy of citizenship, which was previously discussed in this paper, that African Americans in the U.S. face [12].

Groups who suffer stigma may also experience discrimination in the form of being denied healthcare [83]. As discussed, there have been fewer testing sites in African American neighborhoods, suggesting discrimination or bias in testing. Anecdotal and empirical data suggest that African Americans presenting with symptoms of upper respiratory infection (e.g., fever, cough) have been less likely to be tested for COVID-19 compared to Whites, indicating disparate access to immediate testing and treatment [90]. Even after testing positive for COVID-19, many African Americans fear they will not receive life-saving treatment if they suffer severe disease or will be denied care due to discriminatory assumptions about their chances of survival [91].

Fear of contracting COVID-19 is high in African American communities, illustrated by population survey estimates that 33% of African Americans are very concerned they will contract and be hospitalized with COVID-19, compared to only 18% of Whites [91]. African Americans are also twice as likely as Whites to know someone who has been hospitalized with or has died from COVID-19 [92]. This can create second-hand trauma when African Americans continually witness the pain and suffering of people who look like them [91]. These factors warrant greater attention to the role of social determinants of health and stigma in COVID-19.

## 5. Racism, Stigma, COVID-19, and Health: Proposed Integrated Weathering Framework

It is clear that the relationships between racism, stigma, COVID-19, and health outcomes among African Americans are dynamic and multifactorial. Thus, developing a comprehensive theoretical model that illustrates these relationships would have substantial implications for future research, programs, and policies to improve the health of African American communities during this critical time. Versions of Bronfenbrenner’s socio-ecological model [93,94] have been widely used to conceptualize how the multiple nested determinants of health (i.e., biological, behavioral, psychological, sociocultural, medical, environmental, and political factors) interact with each other and contribute to health and disease risk. Further, the life course model of health [95] conceptualizes health as the result of the cumulative effects of both risky and protective factors and events across the lifespan as well as intergenerational factors. The Weathering Hypothesis, originally developed by Geronimus [96] to explain the significant disparities in adverse birth outcomes among African Americans and Whites, expands on both the socio-ecological and life course models to elucidate the etiology of premature mortality and morbidity among African Americans. 

The Weathering Hypothesis theorizes that African Americans experience accelerated biological aging due to cumulative exposures to stressors attributable to adverse social, economic, environmental, and political conditions [97]. This hypothesis further posits that frequent exposures to stressors result in continuous, “high-effort” coping responses at the physiological level, which, in turn, negatively affect health [97], p. 826. Importantly, the Weathering Hypothesis helps explain not only premature mortality and morbidity among African Americans, but also the widening health disparities among African Americans and Whites as age increases. For example, Geronimus’ seminal research discovered that the established curvilinear relationship between maternal age and adverse birth outcomes (i.e., teenagers and older women experiencing a higher risk of unfavorable birth outcomes than women in their 20s) did not hold for African American women; instead, teenage African American mothers experienced better birth outcomes when compared to their older counterparts [96,98,99,100]. Moreover, a systematic review by Forde et al. [98] found widespread empirical evidence supporting the role of weathering in contributing to adverse obstetric outcomes (e.g., decreased infant birthweight, preterm birth, intrauterine growth retardation, infant mortality) and chronic health conditions (e.g., diabetes, hypertension, cardiovascular disease, anemia).

**Figure 1:**
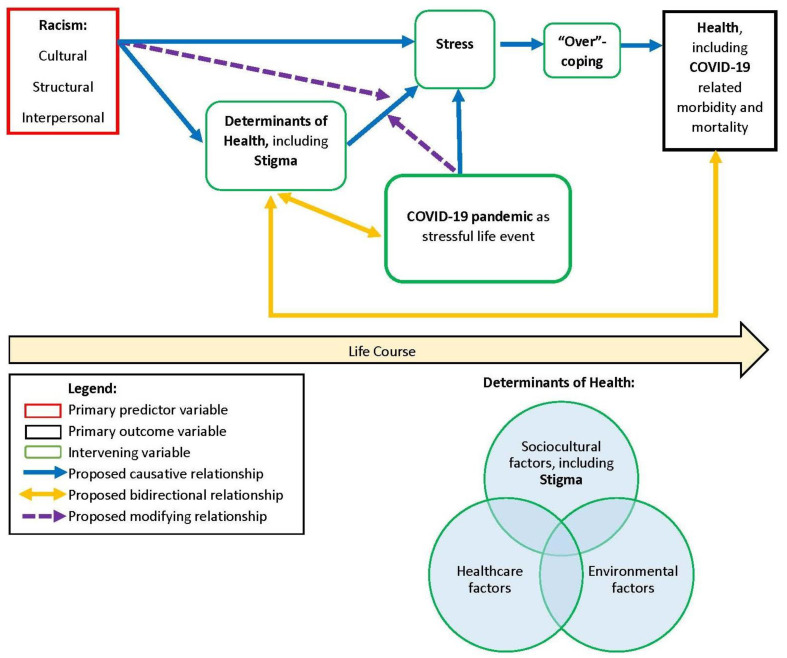
Application of Integrated Weathering Framework: Proposed Relationships between Racism, Stigma, COVID-19 and Health Outcomes Among African Americans.

Overall, the Weathering Hypothesis comprehensively connects the socio-ecological model with the life course model to illustrate the significantly disparate health trajectories that African Americans and Whites experience, and it has also been heavily supported in research studies during the past few decades. Therefore, we propose an integrated Weathering Framework to depict predicted relationships between racism, stigma, COVID-19, and health outcomes among African Americans. In the conceptual diagram (Figure 1), the blue arrows represent proposed causative links, the yellow arrows denote proposed bidirectional relationships, and the purple dotted arrows signify proposed modifying relationships. At the crux of this framework and as substantiated in the literature, racism (including cultural, structural, and interpersonal forms of racism) is a root cause of diminished health and wellbeing among African Americans. We posit that racism operates through, or interacts with, the multiple determinants of health to yield both acute and chronic experiences of stress. In other words, *the determinants of health likely partially mediate the associations between racism and stress*; additionally, *the experience of racism also likely modifies the impacts of negative determinants of health*, thus leading to increased stress. In the proposed framework, we also acknowledge that repeated exposures to *interpersonal racism are directly associated with chronic stress* [22,24].

Based on the socio-ecological model, these multiple determinants of health are inextricably interrelated and are nested within individual, interpersonal, community, and broader systemic factors [93,94]. Specifically, determinants of health comprise sociocultural factors, including socioeconomic status, gender, race, ethnicity, and stigma as well as access to and quality of education, employment, housing, transportation, and food/nutrition; healthcare factors such as quality of, access to, and coordination of healthcare as well as health literacy; and environmental factors, including characteristics of the surrounding physical environment and safety. Our proposed conceptual framework indicates *bidirectional relationships between the determinants of health and health outcomes*. The framework embodies the life course model by emphasizing that the accumulation of risky and protective exposures to these multiple determinants of health over the life course directly impact one’s health outcomes, including COVID-19-related mortality and morbidity. Further, we posit that one’s health status also likely impacts one’s sociocultural, healthcare, and environmental conditions and opportunities, thereby perpetuating the cycle by which the most vulnerable individuals experience worsening outcomes over the life course. For example, individuals with chronic illnesses as well as their families are more likely to experience greater socioeconomic burdens such as higher healthcare expenditures, reduced likelihood of obtaining or sustaining full-time employment, and decreased educational attainment [101,102,103]. 

Moreover, we hypothesize that as individuals experience frequent exposures to acute and chronic stressors, rooted in racism and negative determinants of health, over the life course, they physiologically adapt to their circumstances by “over-coping,” or the high-effort coping described by Geronimus. Research studies have tested the Weathering Hypothesis by exploring various biological mechanisms linking broader determinants of health to disparities in health outcomes. The majority of biomarkers, such as cortisol [104], sympathetic nerve activity [105], blood pressure reactivity [106,107], cytokine production [108], and glycated hemoglobin [109,110], used in these studies are either direct or potential indicators of physiological stress responses [98]. In terms of the next component of our proposed framework, over-coping likely impacts health outcomes through the accumulation of allostatic load, a construct coined by McEwen and Seeman [111]. Allostatic load is broadly described as the “cumulative wear and tear on the body’s systems owing to repeated adaptation to stressors” [97], p. 826. Additionally, based on findings from a multitude of studies, shortened cellular telomere length, a biomarker for aging [112], is significantly associated with chronic physical and mental health deterioration and mortality [113,114,115] as well as stress and stressors (i.e., childhood trauma, stressful life events) [116,117,118,119]. Therefore, the shortening of telomere length is considered to be a potential biological mechanism through which stress and the consequent accumulation of allostatic load impact health outcomes and lead to accelerated aging, per the Weathering Hypothesis. Importantly, many research studies have found that perceived racism and discrimination are significantly associated with shortened telomere length among African Americans [120,121,122,123], thereby further substantiating our proposed Weathering Framework. In addition to the neuroendocrine and immune system pathways that directly link stress and health outcomes, widespread research indicates that exposure to stress may also entail “over-coping” through unhealthy behaviors, including poor diet, tobacco, alcohol and other substance misuse, and risky sexual behaviors, which are, in turn, associated with adverse health outcomes [124,125,126,127,128,129,130,131,132,133]. 

Finally, we posit that COVID-19 plays two key roles in the proposed conceptual framework. First, a COVID-19 diagnosis is a health outcome, and widespread evidence has indicated that African Americans have significantly higher rates of COVID-19 cases as well as greater case-fatalities when compared to Whites [8,9,10]. Second, we envision the COVID-19 pandemic, regardless of disease status, as a *stressful life event that results in experiences of stress* [134,135,136] *and has a bidirectional relationship with the determinants of health, including stigma*. Specifically, pandemic-related effects that are particularly salient among communities of color and other vulnerable populations have included negative determinants of health, such as unemployment or underemployment, increased isolation, reduced educational opportunities, postponement of preventive care needs, reduced access to public transportation as well as free or subsidized meals, and increased stigma related to racial or cultural identity [137,138,139]. Further, we hypothesize that vulnerable individuals who have already experienced chronic exposures to the negative determinants of health over the life course will be more likely to perceive the pandemic as stressful, as they may not have adequate protective resources or conditions to withstand the adverse impacts of the pandemic without experiencing significant health implications in the long term. Along these lines, we also posit that COVID-19, *as a stressful life event, potentially modifies the relationship between the determinants of health and stress*, in that it may render more pronounced impacts among these aforementioned vulnerable individuals, when compared to individuals who are exposed to favorable determinants of health, and place them at greater risk for experiencing both acute and chronic stress. Overall, our Weathering Framework theorizes that COVID-19 not only operates as a potential health outcome but also operates as a critical stressful life event that exacerbates existing physical and mental health disparities, which are rooted in racism and related to the multiple determinants of health.

## 6. Implications and Future Directions

The existence of racism as a root cause of COVID-19 health disparities among African Americans, as described in the proposed Weathering Framework, necessitates the expansion and continuation of programs, clinical interventions, policies, and further research to improve health equity. Public health officials and community leaders can help prevent stigma by using media channels, including social media and news media, to denounce the stereotyping of groups of people who experience stigma because of COVID-19 [83]. Fostering culturally tailored behavioral and mental health dialogue and responses should help promote proactive self-care, reduce stigma, and encourage integrated health care [8]. Developing comprehensive national programs to provide integrated health care to the underinsured and uninsured impacted by COVID-19 would promote increased resiliency within African American communities and reduce their susceptibility to adverse outcomes and long-term socioeconomic hardships [14]. Clinical care providers must be cognizant of the historical mistrust of medical providers and public officials that may increase the emotional needs of African Americans and ensure collaborative treatment to help strengthen patient buy-in [140]. Importantly, in terms of both clinical and programmatic settings, there is a critical need to increase diversity in the workforce, including the incorporation of community health workers, in order to strengthen provider–client relationships and help community residents feel more comfortable, connected, and empowered while obtaining health and human services [20,141,142].

Policy options and action opportunities to close the health equity gap include working towards universal healthcare coverage with particular attention to social and cultural barriers. This can be achieved by expanding the availability of comprehensive primary and secondary services, which address the multiple determinants of health, in minority communities, and by improving coordination between levels of care. Strategies should be tested and evaluated to extend access to and ensure quality care for minority communities, with provisions that stigmatization and inequities are not reinforced. Other strategies include promoting ongoing engagement among multiple sectors to institutionalize health equity goals and collecting data that measures racial inequities in health to provide the foundation for political action and accountability on the determinants of health and the advancement of health equity [143]. Furthermore, addressing health disparities will require a multifaceted approach from diverse stakeholders, including academic institutions [144]. Therefore, solutions are needed to enhance community engagement among stakeholders of African American health, augment population and public health funding to promote health equity among African Americans, and increase the number of African American healthcare providers [80]. These efforts may help mitigate the negative effects of hierarchy of citizenship [12] that exist among communities of color in the U.S.

The proposed conceptual framework also has important implications for future research directions. First, we recommend that longitudinal studies investigate the independent and combined impacts of racism and COVID-19, as a pandemic and as a diagnosis, on long-term mental and physical health outcomes across the life course as well as the societal and biological mechanisms through which these impacts occur. Second, large-scale research studies must link various sources of data, including population-based observational studies, vital statistics, census data, health insurance claims databases, and other administrative data, to simultaneously examine the multiple determinants of health and their complex interactions with COVID-19 among communities of color. Gaining insight into the relationships between racism, COVID-19, and the multiple determinants of health will enable community stakeholders to develop more coordinated responses, comprising strategic partnerships among public, private, health, and non-health-related sectors, to the pandemic; these responses should ideally entail intentionally anti-racist programmatic, clinical and policy interventions as well as long-term systemic changes in the community infrastructure that promote health equity. Importantly, we posit that researchers, program developers, and policymakers can also employ lessons learned from utilizing our proposed integrated Weathering Framework to examine and mitigate the adverse impacts of future population health crises, such as natural disasters, communicable disease epidemics, and environmental health emergencies, among communities of color and other vulnerable populations. 

## 7. Conclusions

The disproportionate impact of COVID-19 on African American communities necessitates a deeper exploration of the intersectional roles of racism, stigma, and other social determinants of health in influencing disease and mortality risk. In this paper, we developed and applied an integrated Weathering Framework to illustrate the dynamic interrelationships between these critical factors and conceptualized COVID-19 as a stressful life event that will have profound health implications over the life course for African Americans. The proposed Weathering Framework, which explores the role of racism as a root cause of COVID-19 health disparities among African Americans, can be employed to inform future population health and clinical interventions, policies, and continued research to improve health equity.

## Data Availability

No new data were created or analyzed in this study. Data sharing is not applicable to this article.
